# Effects of Anti-COVID-19 Vaccination and Pre-Exposure Prophylaxis with Tixagevimab-Cilgavimab in Kidney and Liver Transplant Recipients

**DOI:** 10.3390/medicina59122101

**Published:** 2023-11-30

**Authors:** Roberta Angelico, Francesca Romano, Luigi Coppola, Marco Materazzo, Domiziana Pedini, Maria Sara Santicchia, Roberto Cacciola, Luca Toti, Loredana Sarmati, Giuseppe Tisone

**Affiliations:** 1 HPB and Transplant Unit, Department of Surgical Sciences, University of Rome Tor Vergata, 00133 Rome, Italy; 2 Department of System Medicine, Tor Vergata University, 00133 Rome, Italy; 3 Infectious Disease Clinic, Policlinico Tor Vergata, 00133 Rome, Italy

**Keywords:** SARS-CoV-2, anti-SARS-CoV-2 antibodies’ titer, COVID-19 disease, pre-exposure prophylaxis, long-active antibody, tixagevimab, cilgavimab, solid organ transplantation, kidney transplantation, liver transplantation, immunosuppression

## Abstract

*Background and Objectives*: Underpowered immune response to vaccines against SARS-CoV-2 was observed in solid organ transplant (SOT) recipients. A novel combination of monoclonal antibodies tixagevimab-cilgavimab (TGM/CGM) received authorization as pre-exposure prophylaxis (PrEP) in those with reduced response to vaccine. We aimed to evaluate the response rate to COVID-19 vaccination in kidney transplant (KT), compared to liver transplant (LT) recipients, and the efficacy and safety of PrEP with TGM/CGM. *Material and Methods*: Between March and November 2022, adult KT and LT recipients who had completed the vaccination schedule (3 doses) were tested for anti-SARS-CoV-2 antibodies titer. SOT recipients with anti-SARS-CoV-2 titer ≥ 100 IU/mL were considered protected against infection, while those with titer < 100 UI/mL were defined non-protected. Patients with inadequate response were invited to PrEP. *Results*: In total, 306 patients were enrolled [KT:197 (64.4%), LT:109 (35.6%)]. After the complete scheme of vaccination, 246 (80.3%) patients developed a protective titer, while 60 (19.6%) did not have a protective titer. KT recipients had a lower rate of protective anti-COVID-19 titer compared to LT patients [149 (75.6%) vs. 97 (89.0%), *p* = 0.004]. Recipients with non-protective anti-COVID-19 titer received mainly tacrolimus-based regimen associated with mycophenolate mofetil (MMF) (70%) e steroids (46.7%) as maintenance immunosuppression, while those treated with everolimus were associated with higher protective titer. Of 35 (58.3%) patients who received PrEP, within 12 months, 6 (17.1%) (all KT) developed pauci-symptomatic COVID-19 disease, while 15/25 (60%) of non-responders, who did not receive the prophylaxis, developed COVID-19 disease. After PrEP, hospitalization rate was lower (2.8% vs. 16%), and no adverse events, neither graft loss nor rejection, were observed. *Conclusions*: Despite complete COVID-19 vaccination, SOT recipients might be not protected from the SARS-CoV-2 infection, especially after KT. In non-protected SOT patients, the subsequent pre-exposure prophylaxis with combination of monoclonal antibodies (TGM/CGM) might be an efficacy and safe strategy to prevent COVID-19 severe disease and hospitalization.

## 1. Introduction

The Coronavirus Disease 2019 (COVID 19) pandemic, declared in March 2020 by World Health Organization (WHO), represented a major concern with more than 771 million cases reported, causing almost 7 million deaths worldwide [1]. The high risk of human-to-human transmission and elevated mortality rate among vulnerable populations, such as elderly, individuals with comorbidities or with suppression of the immune system, overwhelmed healthcare systems during the initial pandemic wave until mass immunization through vaccination became available [2,3,4,5]. 

Recipients of solid organ transplant (SOT) face a particularly high risk of severe COVID-19 disease [6] due to the acquired immunosuppressive state and presence of comorbidities [7]; this has led to hospitalization and mortality rates ranging from 26% to 63% [8,9,10] and 13% to 30%, respectively [11,12]. 

However, several preventing and therapeutic strategies including supportive care, oxygen therapy, antibiotics (in case of specific indications), anticoagulant formulations (both enoxaparin and heparin) or low-dose steroids (i.e., Dexamethasone 6 mg/day) were implemented to act against the SARS-CoV-2 pandemic and to improve survival outcomes [13,14,15]. Moreover, in frail categories such as dialytic patients, an early assumption from the onset of symptoms (within 48 h) of antiviral therapy, namely, Remdesivir, a nucleoside analog that inhibits the RNA-dependent RNA polymerase (RdRp) of coronaviruses, has been described to have the potential to shorten the recovery time without any adverse effects [16]. 

However, given the lack of specific antiviral therapy to control the virus’s spread, initially, a worldwide effort was made to develop effective vaccines, leading to the first authorization by the Food and Drug Administration (FDA) and the European Medicines Agency (EMA) in December 2020–January 2021 for the use of nucleoside-modified mRNA vaccines including BNT162b1 (Comirnaty, Pfizer-BioNTech, New York, NY, USA) and mRNA-1273 (Spikevax, Moderna, MA, USA)” [17]. While phase III trials reported high vaccine efficacy in healthy subjects, a fluctuating or underpowered immune response after vaccines against SARS-CoV-2 was observed in SOT recipients, despite their access to vaccination being prioritized [18,19]. Therefore, SOT recipients potentially became a basin of individuals who had represented a continued unintentional viral exposure for the general healthy population [7,20,21], as well as a potential source of new variants [7,22]. 

In response to the risk of breakthrough infections, additional measures were introduced in the SOT population. In December 2021, a novel long-active antibody (LAAB) combination of tixagevimab-cilgavimab (TGM/CGM) (Evusheld^®^, AstraZeneca Pharmaceuticals LP, Wilmington, DE, USA) received authorization by regulatory agencies as pre-Exposure Prophylaxis (PrEP). The access to monoclonal antibodies TGM/CGM has been granted to be administered in patients who may have a reduced immune response to COVID-19 vaccine after two weeks from vaccination or in those who have history of severe adverse reactions to COVID-19 vaccine [23]. In the ACTIV-3 trial, an international randomized-controlled phase III study, authors documented that the use of TGM/CGM was safe and associated with low mortality in hospitalized COVID-19 patients, but it did not significantly improve the time to sustained recovery [24]. Nevertheless, some concerns were raised regarding its effectiveness as PrEP for the new variants of concern, such as the Omicron Variant [25], and lack of data are reported regarding the TGM/CGM’s use as PrEP in SOT recipients.

The present study aims to evaluate the response rate to COVID-19 vaccination and the subsequent efficacy and safety of PrEP, with combined monoclonal antibodies TGM/CGM, in kidney and liver transplantation recipients. 

## 2. Materials and Methods

### 2.1. Study Design

A monocentric, observational, single-armed study named “COVID-19 Transplant” (NCT05629923) was designed in March 2022 at the Liver and Kidney Transplant Unit and Infectious Disease Clinic of the University of Rome Tor Vergata, Rome, Italy. The primary aim of the study was to assess the response rate to COVID-19 vaccination in SOT patients. The secondary aim was to evaluate the efficacy and safety of pre-exposure prophylaxis (PrEP) with tixagevimab-cilgavimab (TGM/CGM, Evusheld^®^, AstraZeneca Pharmaceuticals LP, Wilmington, DE, USA) in SOT recipients not responder to full scheme of vaccination. 

All adult patients who had undergone kidney transplantation (KT) or liver transplantation (LT) with a functioning allograft, transplanted more than three months prior to enrollment, and who had completed the vaccination schedule, were included in the study. The complete vaccination schedule was defined according to the Italian Guidelines, present at the time of the study, as two doses of vaccination plus a booster dose unless any history of recent COVID-19 infection [26]. Exclusion criteria encompassed individuals under 18 years of age, pregnancy, patients experiencing previous biopsy-proven acute rejection (BPAR), patients with SARS-CoV-2 infection at the time of enrollment, and those with a known adverse reaction to COVID-19 vaccination or TGM/CGM. Furthermore, SOT patients with white blood cell abnormalities (leukopenia or leukocytosis), upper respiratory flu-like symptoms (e.g., fever, cough), other active infections (e.g., cytomegalovirus), and those receiving high doses of corticosteroids were also excluded.

Ethical approval was waived by the local Ethics Committee of the University of Rome Tor Vergata, as all the procedures outlined in the study protocol were part of routine care. All procedures were conducted in accordance with ethical standards set by the institutional and national research committees, adhering to the 1964 Helsinki Declaration and its subsequent amendments or comparable ethical standards. Written informed consent, specifically for research purposes, was routinely obtained and renewed after each procedure (e.g., blood sample, PrEP administration).

### 2.2. Outcomes Measurements

For the primary aim, the response rate to COVID-19 vaccination in SOT patients was evaluated by the dosage of the serum titer of antibodies against SARS-CoV-2 using Electrochemiluminescence Immunoassay (ECLIA) (Elecsys Anti-SARS-CoV-2, F. Hoffmann-La Roche AG, Basel, Switzerland). A protective anti-COVID-19 serological titer was defined as ≥100 I.U./mL.

The efficacy of TGM/CGM was assessed by (1) the incidence of COVID-19 infections within 12 months from PrEP; (2) a protective anti-COVID-19 serological titers after 12 months from PrEP. Safety was defined according to the occurrence of adverse effects after TGM/CGM administration including cardiovascular and thrombo-embolic events, and clinically significant hemorrhagic events. To evaluate the secondary aim, KT and LT recipients were further sub-analyzed according to the presence or absence of protective anti-COVID-19 serological titers.

### 2.3. Pre-Exposure Prophylaxis with Tixagevimab-Cilgavimab

After obtaining informed consent, KT and LT recipients were enrolled during their routine outpatient follow-up visit. At the first visit, anti-COVID-19 serum antibodies were measured. Also, biochemical blood tests and nasopharyngeal/oropharyngeal swabs, to detect asymptomatic COVID-19 infections, were performed. If patients showed an inadequate response to COVID-19 vaccination, considered as the presence of non-protective serological anti-COVID-19 titers (<100 I.U./mL), they were invited to undergo PrEP. In such cases, PrEP was administered using intravenous combination of tixagevimab and cilgavimab (TGM/CGM) (Evusheld^®^, AZD7442, AstraZeneca Pharmaceuticals LP, Wilmington, DE, USA), and each antibody was given individually within 30 min. All participants were monitored for one hour after administration, and any adverse events were recorded. A follow-up via phone was scheduled one week later for all patients who had received PrEP to report any side effects.

Regardless of their immunological status towards COVID-19, after 12 months from enrolment, a follow-up visit was conducted to identify any COVID-19 cases during the observation period, gathering information about symptoms, hospitalization, and intensive care unit admissions.

**Figure 1:**
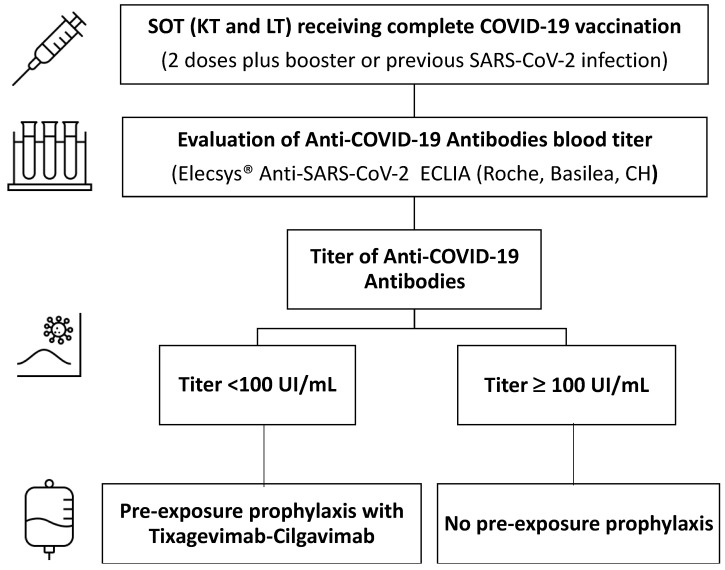
Study protocol for pre-exposure prophylaxis with Tixagevimab-Cilgavimab in solid organ transplant recipients. Abbreviations: COVID-19, Coronavirus disease 19; SOT, solid organ transplantation.

In patients who received PrEP, an additional anti-COVID-19 titer measurement was planned using ECLIA (Elecsys Anti-SARS-CoV-2, F. Hoffmann-La Roche AG, Basel, Switzerland) during their routine follow-up visit at 12 months from enrollment. Figure 1 summarizes the study protocol.

### 2.4. Data Collection

Patients’ demographic characteristics, transplant variables, and immunosuppressive regimen data were collected retrospectively from a maintained perspective database of SOT patients undergoing follow-up at our facility. SOT patients’ demographics included age at transplant, gender, body mass index (BMI), preoperative comorbidities, previous oncological diagnoses, and Charlson Comorbidity Index (CCI). Transplant data included type of SOT, indication to transplant, and peri- and post-operative immunosuppressive regimen. For LT recipients, the Model for End-Stage Liver Disease (MELD) score at transplant was also recorded. For KT, additional data included blood group and graft/transplant features (single or dual, re-transplantation). Immunosuppressive regimens were analyzed at discharge and at the time of enrollment in the study.

### 2.5. Transplant Immunosuppressive Regimens

In our center, the immunosuppression regimen for LT is a steroid-free approach ab initio and is based on the use of calcineurin inhibitors (CNIs), usually with tacrolimus (TAC) while less frequently with cyclosporine (CsA), in combination with mammalian target or rapamycin (m-TOR) inhibitors—everolimus (EVR). Within the first 3 months after LT, the standard target trough blood levels are set at 3–5 ng/mL for TAC, 700–800 ng/mL for CsA two hours after its oral intake (CsA t2), and 3–6 ng/mL for EVR. Thereafter, the targets for LT are 2–4 ng/mL for TAC, t2 600–700 ng/mL for CsA t2, and 2–4 ng/mL for EVR. In case of concomitant renal dysfunction, delayed CNIs introduction (e.g., to fourth postoperative day after LT) is adopted as long as induction therapy with interleukin-2 antagonist receptor (IL-2RA), such as basiliximab, is administered at the time of transplantation and in post-operative day 4 in order to maintain early immunosuppression efficacy.

Immunosuppressive regimen for KT is based at our institution on tacrolimus (TAC) once daily (0.15 mg/kg/day), mychophenolate mofetil (MMF) (500–1500 mg/day) or sodium (360–1440 mg/day), and steroids (20 mg/day tapered to 5 mg/day within 3 months). Depending on panel reactive antibody (PRA) score, induction therapy at the time of KT can be adopted with the use of basiliximab (20 mg intraoperatively and on the fourth postoperative day) or antithymocyte globulin (ATG) (1.5 mg/kg/day for the first three postoperative days). For KT, a tacrolimus trough level of 7–9 ng/mL is aimed for the first month after KT, along with 6–8 mg/mL within 6 months, and 5–6 ng/mL thereafter.

### 2.6. Statistical Analysis

All data were collected using Microsoft Excel software version 16.77.1 2023 Microsoft. Statistical analysis was performed using IBM SPSS 26.0 software. Continuous variables were reported as means and standard deviations. Normally distributed continuous data were analyzed using a parametric test (Student’s *t*-test), and non-normally distributed data were analyzed using the Mann–Whitney U test. Categorical variables were reported as frequencies and percentages, and χ^2^ tests or Fisher’s exact tests were applied based on sample size. A *p*-value of less than 0.05 was considered significant.

## 3. Results

### 3.1. Study Population

From 1 March 2022, to 30 November 2022, 328 SOT patients were considered for enrollment. Of these, 306 (93.3%) SOT patients were included in the analysis, while 22 (6.7%) patients were excluded (13 recipients received transplantation from less than 3 months, 5 patients had tested positive for anti-COVID-19 prior to enrollment, 2 recipients were treated for graft rejection with high doses of corticosteroids, 2 patients refused to be included).

Among included SOT patients, 197 (64.4%) patients received KT and 109 (35.6%) underwent LT. Table 1 summarizes demographic variables of the study population. The mean age at enrollment was 58.72 ± 11.98 years, being similar between LT and KT recipients, while the male gender was more frequent in the LT group [LT: 86 (78.9%) vs. KT: 116 (58.9%), *p* = 0.0004]. Sixty-one (19.9%) recipients had a personal history of neoplasm, and this was more frequent in LT recipients compared to KT recipients [LT: 51 (46.8%) vs. KT: 10 (5.1%), *p* < 0.0001], with a mean calculated CCI of 5.31 ± 1.78.

Regarding immunosuppressive regimen, induction agents were used in 117 (38.2%) at transplantation, mainly in KT recipients [112 (56.9%) vs. 5 (4.6%)]. At study enrolment, the overall mean time from transplantation was 5.24 ± 5.62 years and most SOT patients received a TAC-based immunosuppressive regimen (81.3%), equally distributed among KT and LT recipients. As a second drug, KT received mainly MMF [KT: 155 (78.7%) vs. LT: 11 (10.1%), *p* < 0.0001], while EVR was more frequently used in LT [KT: 33 (16.8%) vs. LT: 79 (72.5%), *p* < 0.0001]. Steroids were administrated only in 111 (56.3%) KT recipients, while they were not given in LT patients (*p* < 0.0001).

### 3.2. COVID-19 Vaccination Immune Response

At enrolment, 148 (48.3%) SOT recipients had experienced a previous COVID-19 infection, with 22 (7.18%) being hospitalized and 9 (2.5%) requiring oxygen therapy during admission, without differences between KT and LT recipients. A total of 304 (99.3%) patients received a complete vaccine cycle, and 14 (4.58%) SOT recipients did not receive a booster vaccination due to COVID-19 infection in the prior 4 months. Immunization was obtained solely with nucleoside-modified mRNA vaccines, as per guidelines for the frail population [27]. In 305 (99.67%) SOT recipients, BNT162b1 (Comirnaty, Pfizer-BioNTech, New York, USA) was preferred, and only one SOT recipient completed the vaccination course with mRNA-1273 (Spikevax, Moderna, Massachusetts, USA).

Overall, 246 (80.4%) patients presented a protective COVID-19 titer (≥100 IU/mL), while 60 (19.6%) patients did not have a protective COVID-19 titer. The mean anti-COVID-19 titer was 1644 ± 1081 IU/mL. KT recipients presented less protective titer compared to LT (KT: 149 (75.6%) vs. LT: 97 (89%), *p* = 0.004). The mean antibodies titer against SARS-CoV-2 was higher in LT compared to KT [KT: 1647 (1080.1) vs. LT: 1657.1 (1076.9), *p* = 0.01]. Patients with anti-COVID-19 protective title received mainly EVR, while fewer were treated with MMF and steroids as detailed in Table 2. Among each group, KT and LT patients who had protective and non-protective anti-COVID-19 titers had similar characteristics (Table 3 and Table 4).

### 3.3. Outcomes of Patients without Protective Anti-COVID-19 Titer

Out of 60 (19.6%) SOT patients with non-protective anti-COVID-19 titers, defined as “low-responders” to anti-SARS-CoV-2 vaccination, 35 (58.3%) received PrEP with TGM/CGM. Of these, none of them developed adverse events after TGM/CGM infusion. At 12 months of follow-up, 6 (17.1%) patients (all KT recipients) developed COVID-19 disease, including 5 (83.3%) patients with pauci-symptomatic infection and 1 (2.8%) patient who required hospitalization. No graft loss or acute rejection were observed.

Thus, 25 (41.7%) patients did not receive infusion of TGM/CGM due to patients’ refusal (*n* = 10) or non-availability of the medication for transplanted patients (*n* = 15), due to prioritization of the available doses to other frail patients such as those immunocompromised for hematological diseases. Of this group, after 12 months of follow-up, 15 (60%) patients (12 KT and 3 LT recipients) developed COVID-19 related-disease, requiring hospitalization in 4 (16%) cases. Table 5 summarizes clinical features and management of those SOT recipients who develop COVID-19 after vaccination.

**Figure 2:**
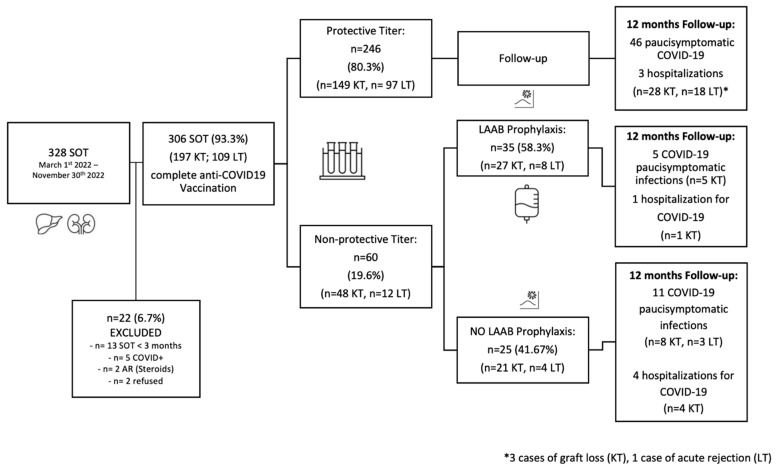
Outcomes of solid organ transplant recipients after anti-COVID-19 vaccination and pre-exposure prophylaxis with long-active antibodies (Tixagevimab-Cilgavimab). Abbreviations: LT, liver transplant; KT, kidney transplant; LAAB, long-active antibodies.

After 12 months from TGM/CGM prophylaxis, in 18 (51.4%) patients, anti-SARS-CoV-2 serum antibodies were investigated and 17 (94.4%) of them reached a protective titer (>100 UI/mL), with a mean titer of 970.4 ± 801.5 UI/mL. Figure 2 summarizes outcomes of SOT recipients after anti-COVID-19 vaccination and PrEP.

### 3.4. Outcomes of Patients with Protective Anti-COVID-19 Titer

After vaccination, 246 (80.4%) SOT patients were found with protective tiles of antibodies for SARS-CoV-2 infection; therefore, they did not receive any prophylaxis with monoclonal antibodies. After 12 months of follow-up, 46 (18.7%) patients (28 KT and 18 LT) experienced COVID-19 disease. In most of the cases (*n* = 43, 93.5%), patients had a pauci-symptomatic disease, while three (1.2%) patients required hospitalization. Three KT recipients with COVID-19 experienced graft loss, while one LT recipient developed acute rejection. No cases of death were reported.

## 4. Discussion

Vaccines preventing SARS-CoV-2 infection have been the most important and truly effective strategy for managing the widespread of the pandemic. Transplant recipients have been considered eligible for vaccination, unless contraindicated from other reasons [7,28]. However, several studies have documented an unpowered immune response of SOT recipients to mRNA vaccination [7,29,30]. Boyarsky et al. reported that a protective antibody response rate was reached only in 54% SOT recipients after the second dose of an mRNA COVID-19 vaccine, being significantly lower compared to the healthy control group [31]. Therefore, based on different kinetics of the antibody response over time in SOT patients, the administration of a third dose of mRNA COVID-19 vaccine has been introduced, increasing antibody response from 6.4 to 69.2% [32,33], with conversions to seropositivity ranging between 33% to 44% rates in those which had been tested as seronegative before the third dosage [6,34]. These results have been confirmed also in a randomized trial comparing rates of protective antibody response (defined as at least 100 UI/mL) in SOT recipients receiving a third dose of mRNA-1273 vaccine versus placebo, obtaining a higher immunogenicity in patients treated with a third dose of vaccination (55% vs. 18%) [35]. The positive effects of additional booster of vaccination have been reported especially in KT recipients, with a response reaching 50–60% [36,37].

In the current study, we analyzed the efficacy of a complete scheme of COVID-19 vaccination (2 doses+ booster or recent COVID-19 infection) and its integration with pre-exposing prophylaxis with combined monoclonal antibodies (TGM/CGM) in kidney and liver transplant recipients “not responder” to vaccination. We observed that the COVID-19 vaccination with booster dose achieved an overall response rate of 80% in our SOT population, being slightly superior in LT recipients (89%) compared to KT patients (75.6%). These data are in line with those reported by Furian et al. (MFI: 19,617 in LT recipients vs 6056 in LT recipients, *p* < 0.001) [38]. Although this difference could be independently affected by the kind of transplanted organ, it might be largely explained by the higher immunosuppression levels in KT recipients, which might affect a lower immune response to vaccination. In KT, we adopt a higher dose of double or triple immunosuppressive drugs (often with steroids) compared to LT recipients, who receive instead a low dose of one or two immunosuppressive drugs. This is also evident by the fact that immunosuppressive regimen modulation in SOT patients is a key factor for the outcome of these developing COVID-19 disease [32,39].

In our study, SOT recipients, who have reached a protective titer (above 100 UI), have more frequently received induction therapy at transplantation and maintenance therapy with everolimus, while a lower response rate was observed in these treated with mycophenolate e corticosteroids. Our results are in line with Netti et al. who reported that therapy with mTOR-inhibitors, which are traditionally able to improve both quantity and quality of memory CD8+ T cells induced by viral infection and vaccination [40], allowing both better humoral and T cell-mediated immune response to COVID-19 vaccination in KT recipients [41]. Also, inhibitors of calcineurin, which represent the main immunosuppressive regimen after SOT, seem to hinder the proliferation of anti-spike antibodies and their levels should be tailored to the patients’ need [42].

During the new incoming Omicron’s dominance (BA2/BA.2.12.1/BA.4/BA.5, March 2022–August 2022) the monovalent vaccines have resulted in reduced efficacy (32% after 90 days from the third dose and 43% after 7 days from the fourth dose) against COVID-19 related hospitalizations in immunocompromised subjects and further strategy have been proposed to protect SOT recipients [43]. These are mainly based on the use of long-active antibody (LAAB) combination with tixagevimab and cilgavimab (TGM/CGM). In 2022, the U.S. Food and Drug Administration approved TGM/CGM combination [44] as its use seems to be effective to provide protection in those who are likely to have a reduced immune response to COVID-19 vaccination or for those who have a history of severe adverse reactions to a COVID-19 vaccine for whom (re)vaccination is not recommended [45]. Pre-exposure prophylaxis with TGM/CGM was not intended as a substitute for vaccination and all subjects should have received a full vaccine series, including booster doses, at least 2 weeks before, unless full vaccination was contraindicated [23]. Although the role of LAAB prophylaxis has been justified for providing an additional level of protection in many immunocompromised patients [46], their use is still debated in the literature and numerous studies seek to confirm their efficacy. Most of the first data were largely based on in vitro evaluations, which were performed before the advent of the Omicron variant. In fact, in vitro research has predicted that TGM/CGM was less protective against that new Omicron variant [47]. Therefore, in SOT recipients who were non-responders to standard vaccination, other protective measures against SARS-CoV-2, such as physical distancing and facemasks, have been suggested to be prioritized, before the efficacy of TGM/CGM against Omicron has been proven [25]. Furthermore, several concerns regarding potential adverse effects of LAAB have been raised: in the PROVENT trial (Phase III Double-blind, Placebo-controlled Study of AZD7442 for Pre-exposure Prophylaxis of COVID-19 in Adult), a higher proportion of subjects who have received TGM/CGM (0.6%) versus placebo (0.2%) reported cardiac adverse effects (myocardial infarctions, arrhythmias, and cardiac failures), although all of these individuals had cardiac risk factors and/or a history of cardiovascular disease at baseline [23,44]. Contrary, in the STORM CHASER (Phase III Double-blind, Placebo-controlled Study of AZD7442 for Post- Exposure Prophylaxis of COVID-19 in adults) trial of post-exposure prophylaxis with TGC/CGM, no cardiac events were noted, although the cohort was younger and had fewer cardiac risk factors [23]. Thus, the benefit of additional protection for immunocompromised patients seemed to outweigh overall the potential risk for cardiac events [23].

In our investigation, the use of LAAB prophylaxis in KT and LT recipients, without protective anti-COVID-19 titers, seemed to be effective and safe. Out of 35 patients who received PrEP with LAAB, only 17.1% patients (all KT recipients) developed COVID-19 disease, being comparable to these who had a “protective” antibody titer after vaccination (18.7%). Contrary, this figure was higher in SOT patient’s “non-responder” to vaccination without receiving the prophylaxis, who developed COVID-19 diseases in up to 41.7% of cases. SOT patients, who were previously treated by prophylaxis with TGC/CGM and experienced subsequent COVID-19 disease, were mainly pauci-symptomatic and had less chance to require hospitalization. Furthermore, after LAAB administration, we did not observe any adverse events neither graft loss nor rejection.

Another interesting piece of data is that out of 18 (51.4%) patients who had evaluated the antibody titles after TCG/CGM prophylaxis, 17 (94.4%) had increased their anti-spike titer above 100 IU/mL, which is considered protective against SARS-CoV-2 infection.

This study has several limitations. Firstly, the low sample size of patients receiving PrEP with LAAB due to the complex clinical condition of transplant recipients and the patients’ reluctance to accept new therapies for SRAS-CoV-2 infection. To overcome this limitation, it is essential that similar studies are performed in order to enlarge the sample and make the evidence more robust. Secondary, KT and LT are two heterogenous population which cannot be compared by many aspects. However, we believe that the comparison of their outcomes might be useful for clinicians to better evaluate the anti-COVID-19 immune response in such fragile SOT categories [48].

## 5. Conclusions

Our study confirms that a complete scheme of anti-SARS-CoV-2 vaccination is essential in patients who underwent organ transplantation. Despite full COVID-19 vaccination being administrated, SOT patients, as immunocompromised patients, might have a reduced antibody response and could be “non-protected” from the SARS-CoV-2 infection, especially after KT due to higher dose of immunosuppressive drugs. In SOT recipients not responding to full scheme of COVID-19 vaccination, subsequent pre-exposure prophylaxis with combination of monoclonal antibodies, such as TGM and CGM, might be safe and effective to reach a protective anti-SARS-CoV-2 antibody titers and, consequently, to prevent COVID-19 severe disease and hospitalization.

## Figures and Tables

**Table 1:** Clinical Variables of the study population.

Variables	Total N (%) or Mean ± SD	Kidney Transplant N (%) or Mean ± SD	Liver Transplant N (%) or Mean ± SD	*p*-Value *
**Number**	306 (100%)	197 (64.4%)	109 (35.6%)	**--**
**Demographic variables**				
**Gender (male)**	202 (66.0%)	116 (58.9%)	86 (78.9%)	**0.0004**
**Age at SOT (years)**	53.15 (12.60)	53.17 (12.57)	53.11 (12.62)	0.999
**Time since sot (years)**	5.24 (5.62)	5.25 (5.63)	5.26 (5.63)	0.756
**BMI**	24.7 (4.2)	24.2 (4.1)	25.6 (4.3)	0.004
**Previous neoplasia**	61 (19.9%)	10 (5.1%)	51 (46.8%)	**<0.0001**
**CCI**	5.31 (1.78)	4.69 (1.19)	6.39 (2.08)	0.579
**Induction therapy at SOT**	117 (38.2%)	112 (56.9%)	5 (4.6%)	**<0.0001**
**Data at enrolment**				
**Age at enrolment (years)**	58.72 (11.98)	57.5 (12.35)	60.96 (10.97)	0.016
**Immunosuppression:**				
**-Tacrolimus**	249 (81.3%)	166 (84.3%)	83 (76.1%)	0.092
**-Everolimus**	112 (36.6%)	33 (16.8%)	79 (72.5%)	**<0.0001**
**-MMF**	166 (54.2%)	155 (78.7%)	11 (10.1%)	**<0.0001**
**-Steroids**	111(36.27%)	111 (56.3%)	0 (0%)	**<0.0001**
**Past personal history of COVID-19**	148 (48.3%)	98 (49.74%)	50 (45.9%)	0.551
**Anti-COVID-19 protective titer**	246 (80.4%)	149 (75.6%)	97 (89.0%)	**0.004**
**Anti-COVID-19 titer (UI)**	1644.0 (1081)	1647 (1080.1)	1657.1 (1076.9)	**0.01**

* *p* value is calculated between liver and kidney transplant recipients. Abbreviations: BMI, body mass index; MMF, mycophenolate mofetil; SOT, solid organ transplantation; SD, standard deviation; CCI, Comorbidity Charlson Index. Bold is the underline parameters and significance of the statistical tests.

**Table 2:** Clinical characteristics of solid organ transplant recipients with protective and non-protective anti-COVID-19 antibody titers.

Variables	Total N (%) or Mean ± SD	Protective Titer N (%) or Mean ± SD	Non-Protective Titer N (%) or Mean ± SD	*p*-Value *
**Number**	306 (100%)	246 (80.3%)	60 (19.6%)	
**Demographic variables**				
**Gender (male)**	202 (66.0%)	168 (68.3%)	34 (56.7%)	0.096
**Type of transplant KT/LT**	197 (64.4)/109 (35.6)	149 (60.5)/97 (39.4)	48 (80)/12 (20)	
**Age at SOT (years)**	53.15 (12.60)	53.17 (11.73)	53.33 (12.65)	0.964
**Time since SOT (years)**	5.24 (5.62)	5.13 (5.64)	5.95 (5.5)	0.316
**BMI**	24.7 (4.2)	24.8 (4.3)	24.6 (4.9)	0.007
**Previous neoplasia**	61 (19.9)	52 (21.1)	9 (15)	0.368
**Induction therapy at SOT**	117 (38.2)	86 (34.9)	31 (51.7)	**0.025**
**Data at enrolment**				
**Age at enrollment (years)**	58.72 (11.98)	58.55 (11.76)	59.52 (12.91)	0.575
**Immunosuppression:**				
**-Tacrolimus**	249 (81.3%)	201 (81.7%)	48 (80%)	0.716
**-Everolimus**	112 (36.6%)	98 (39.8%)	14 (23.3%)	**0.017**
**-MMF**	166 (54.2%)	124 (50.4%)	42 (70%)	**0.008**
**-Steroids**	111 (36.27%)	83 (33.8%)	28 (46.7%)	**0.072**

* *p* value is calculated between patients with protective and non-protective anti-COVID-19 antibodies titers. Abbreviations: BMI, body mass index; MMF, mycophenolate mofetil; SOT, solid organ transplantation; SD, standard deviation. Bold is the underline parameters and significance of the statistical tests.

**Table 3:** Clinical characteristics of kidney transplant recipients with protective and non-protective anti-COVID-19 antibody titers.

KT Variables	Total N (%) or Mean ± SD	Protective Titer N (%) or Mean ± SD	Non-Protective Titer N (%) or Mean ± SD	*p*-Value *
**Number**	197 (100%)	149 (48.7%)	48 (15.7%)	
**Demographic variables**				
**Gender (male)**	116 (37.9%)	92 (61.7%)	34 (56.7%)	0.301
**Serum Creatinine (mg/mL)**	1.89 (1.03)	1.88 (1.05)	1.95 (0.97)	0.691
**Age at SOT (years)**	53.17 (12.56)	53.16 (11.73)	53.24 (12.56)	0.261
**Time since SOT (years)**	5.25 (5.63)	5.13 (5.64)	5.95 (5.5)	0.317
**BMI**	24.2(4.1)	24.8 (4.3)	23.6 (4.9)	0.077
**Previous neoplasia**	10 (3.27)	6 (4.02)	4 (8.33)	0.261
**Induction therapy at SOT**	112 (36.6)	82 (55.03)	30 (62.5)	0.405
**Data at enrolment**				
**Age at enrollment (years)**	58.73 (11.98)	58.55 (11.77)	59.52 (12.91)	0.576
**Immunosuppression:**				
**-Tacrolimus**	166 (54.2)	127 (85.3)	39 (81.25)	0.501
**-Everolimus**	33 (16.75)	27 (18.12)	6 (12.5)	0.505
**-MMF**	155 (78.68)	116 (77.85)	39 (79.59)	0.689
**-Steroids**	111 (56.34)	83 (55.70)	28 (58.33)	0.867

* *p* value is calculated between patients with protective and non-protective anti-COVID-19 antibodies titers. Abbreviations: BMI, body mass index; KT, kidney transplantation; MMF, mycophenolate mofetil; SOT, solid organ transplantation; SD, standard deviation. Bold is the underline parameters and significance of the statistical tests.

**Table 4:** Clinical characteristics of liver transplant recipients with protective and non-protective anti-COVID-19 antibody titers.

LT Variables	Total N (%) or Mean ± SD	Protective Titer N (%) or Mean ± SD	Non-Protective Titer N (%) or Mean ± SD	*p*-Value *
**Number**	**109 (100%)**	**97 (88.9%)**	**12 (11%)**	**--**
**Demographic variables**				
**Gender (male)**	86 (78.99%)	76 (69.72%)	10 (9.17%)	1
**MELD Score**	14.85 (6.53)	14.85 (6.53)	14.78 (6.43)	0.422
**Age at SOT (years)**	53.17 (12.56)	53.16 (11.73)	53.24 (12.56)	0.346
**Time since SOT (years)**	5.26 (5.63)	5.17 (5.71)	5.98 (5.52)	0.317
**BMI**	24.7 (4.2)	24.7 (4.2)	24.7 (4.2)	0.676
**Previous neoplasia**	51 (46.7)	44 (45.3)	7 (58.3)	0.542
**Induction therapy at SOT**	5 (4.59)	4 (4.12)	1 (8.33)	0.448
**Data at enrolment**				
**Age at enrollment (years)**	58.73 (11.98)	58.59 (11.79)	59.52 (12.91)	0.589
**Immunosuppression:**				
**-Tacrolimus**	83 (86.14%)	74 (72.29%)	9 (75%)	1
**-Everolimus**	79 (81.44%)	71 (73.19%)	8 (66.67%)	0.733
**-MMF**	11 (10.1%)	8 (8.25%)	3 (25%)	0.102
**-Steroids**	0 (0%)	0 (0%)	0 (0%)	1

* *p* value is calculated between patients with protective and non-protective anti-COVID-19 antibodies titers. Abbreviations: BMI, body mass index; LT, liver transplantation; MMF, mycophenolate mofetil; SOT, solid organ transplantation; SD, standard deviation. Bold is the underline parameters and significance of the statistical tests.

**Table 5:** Clinical characteristics of solid organ transplant recipients who develop COVID-19. Bold is the underline parameters and significance of the statistical tests.

COVID+	Total N (%) or Mean ± SD	Protective Titer N (%) or Mean ± SD	Non Protective Titer Prep Yes N (%) or Mean ± SD	Non Protective Titer Prep No N (%) or Mean ± SD
**Number**	**70 (100%)**	**49 (70%)**	**6 (8.6%)**	**15 (21.4%)**
**Parameters**				
**Age years**	61 (±11.9)	61 (±12)	61 (±11.7)	61 (±12.1)
**Gender (male)**	48 (68.6%)	38 (77.5%)	4 (66.6%)	6 (40%)
**Fever**	66 (94.2%)	47 (95.9%)	6 (100%)	13 (86.6%)
**Cough**	61 (87.1%)	44 (89.8%)	3 (50%)	14 (93.3%)
**Fatigue**	70 (100%)	49 (100%)	6 (100%)	15 (100%)
**Anorexia**	15 (21.4%)	10 (20.4%)	1 (16.7%)	4 (26.7%)
**Myalgia**	59 (84.3%)	47 (95.9%)	2 (33.3%)	10 (66.7%)
**Others**	66 (94.3%)	49 (100%)	4 (66.6%)	13 (86.6%)
**Dyspnea–mild**	54 (77.1%)	40 (81.6%)	5 (83.3%)	9 (60%)
**Dyspnea–moderate**	8 (11.4%)	6 (12.2%)	0 (0%)	2 (13.3%)
**Dyspnea–severe**	8 (11.4%)	3 (6.1%)	1 (16.6%)	4 (26.7%)
**Hospitalization**	8 (11.4%)	3 (6.1%)	1 (16.7%)	4 (26.7%)
**-Remdesevir 200 mg (load)** **+100 mg (4 days)**	8 (11.4%)	3 (6.1%)	1 (16.6%)	4 (26.7%)
**-Dexamethason 6 mg/die**	8 (11.4%)	3 (6.1%)	1 (16.6%)	4 (26.7%)
**-Sotrovimab**	8 (11.4%)	3 (6.1%)	1 (16.6%)	4 (26.7%)
**-Molnupiravir 800 mg/die**	62 (88.6%)	46 (93.8%)	5 (83.3%)	11 (73.3%)
**-Nirmatrelvir 400 mg/die**	62 (88.6%)	46 (93.8%)	5 (83.3%)	11 (73.3%)
**-Ritronavir 100 mg/die**	62 (88.6%)	46 (93.8%)	5 (83.3%)	11 (73.3%)

## Data Availability

The data presented in this study are available on request from the corresponding author. The data are not publicly available due to privacy.

## Abbreviations

COVID 19	The Coronavirus Disease 2019
EMA	European Medicines Agency
FDA	Food and Drug Administration
KT	kidney transplantation
LT	liver transplantation
WHO	World Health Organization
SOT	solid organ transplantation
TGM/CGM	tixagevimab-cilgavimab
PrEP	pre-Exposure Prophylaxis
MMF	mychophenolate mofetil
CNIs	calcineurin inhibitors
TAC	tacrolimus
CsA	cyclosporine
EVR	everolimus
